# Challenges in estimating effective population sizes from metagenome-assembled genomes

**DOI:** 10.3389/fmicb.2023.1331583

**Published:** 2024-01-05

**Authors:** Xiaojun Wang, Xiaoyuan Feng

**Affiliations:** ^1^Shenzhen Research Institute of the Chinese University of Hong Kong, Shenzhen, China; ^2^State Key Laboratory of Lake Science and Environment, Nanjing Institute of Geography and Limnology, Chinese Academy of Sciences, Nanjing, China

**Keywords:** metagenomics, effective population size, microbial evolution, natural selection, genetic drift

## Abstract

Effective population size (*N_e_*) plays a critical role in shaping the relative efficiency between natural selection and genetic drift, thereby serving as a cornerstone for understanding microbial ecological dynamics. Direct *N_e_* estimation relies on neutral genetic diversity within closely related genomes, which is, however, often constrained by the culturing difficulties for the vast majority of prokaryotic lineages. Metagenome-assembled genomes (MAGs) offer a high-throughput alternative for genomic data acquisition, yet their accuracy in *N_e_* estimation has not been fully verified. This study examines the *Thermococcus* genus, comprising 66 isolated strains and 29 MAGs, to evaluate the reliability of MAGs in *N_e_* estimation. Despite the even distribution across the *Thermococcus* phylogeny and the comparable internal average nucleotide identity (ANI) between isolate populations and MAG populations, our results reveal consistently lower *N_e_* estimates from MAG populations. This trend of underestimation is also observed in various MAG populations across three other bacterial genera. The underrepresentation of genetic variation in MAGs, including loss of allele frequency data and variable genomic segments, likely contributes to the underestimation of *N_e_*. Our findings underscore the necessity for caution when employing MAGs for evolutionary studies, which often depend on high-quality genome assemblies and nucleotide-level diversity.

## 1 Introduction

Natural selection and genetic drift serve as two primary mechanisms driving the genetic variability in natural populations. Natural selection acts to favor advantageous alleles and purge deleterious variants, while genetic drift functions through stochastic processes (Kirchberger et al., 2020). The interplay between these forces is largely influenced by the effective population size (*N_e_*), a parameter that characterizes the size of an idealized population, with nonoverlapping generations that has the same specified genetic properties as in the observed population (Charlesworth, 2009; Crow, 2017). Elevated *N_e_* values enhance the efficiency of natural selection, whereas reduced *N_e_* amplifies the impact of neutral drift (Batut et al., 2014). Initially proposed for eukaryotic populations (Wright, 1931), the concept of *N_e_* has been further nuanced into contemporary *N_e_* and long-term *N_e_*, each addressing distinct temporal scales (Hare et al., 2011). Contemporary *N_e_* pertains to the most recent generations and is frequently employed in agricultural breeding and wildlife conservation (Nadachowska-Brzyska et al., 2022). In contrast, microbial research predominantly focuses on long-term *N_e_*, a harmonic mean calculated over a continuous lineage since the most recent common ancestor. This long-term *N_e_* (hereafter *N_e_*) has been implicated in microbial mutation rates (Lynch et al., 2016) and pangenomic evolution (Andreani et al., 2017; McInerney et al., 2017). Additionally, *N_e_* plays a critical role in shaping microbial genome sizes. For example, free-living bacteria with small genomes, such as high-light adapted II (HLII) *Prochlorococcus*, are postulated to possess an extraordinarily large *N_e_* (e.g., 10^9–13^), thereby facilitating their genome reduction process through strong natural selection (Giovannoni et al., 2014). However, our recent study challenges this hypothetical large *N_e_* and reports a substantially smaller *N_e_* of approximately 10^7^, highlighting the significance of genetic drift in microbial genome reduction (Chen et al., 2021). *N_e_* directly affects the core assumptions made by researchers when understanding microbial evolution, so estimating *N_e_* is fundamental for microbial evolution research.

In microbial population analyses, *N_e_* can be calculated using the formula π_s_ = 2 × *N_e_* × μ, where μ represents the spontaneous mutation rate and π_s_ denotes the neutral genetic diversity. However, the direct calculation of *N_e_* presents several challenges: (i) the determination of mutation rates via mutation accumulation experiments is time-consuming and restricted to specific microbial lineages amenable to grow on solid media; (ii) the estimation of neutral genetic diversity hinges on the availability of panmictic populations, which require a large number of closely related genomes (Luo et al., 2017). While mutation rates have been measured for over 35 bacterial and archaeal lineages, comprehensive genomic datasets for panmictic populations are extant for only 22 of these lineages (Chen et al., 2021). These limitations are likely attributed to the inherent difficulties in culturing a majority of prokaryotic lineages (Rinke et al., 2013) and the lack of targeted isolation efforts for required panmictic populations, thereby hindering the *N_e_* estimation for both existing and future lineages with mutation rate available. In the absence of suitable genomic datasets, *N_e_* estimation risks becoming potentially inaccurate.

Metagenomic sequencing provides a high-throughput alternative for obtaining microbial genomic information from environmental samples (Nayfach et al., 2020). This approach has been employed in various evolutionary studies and has demonstrated performance comparable to that of isolates (Anderson et al., 2017; Ngugi et al., 2023). Nonetheless, the reliability of metagenome-assembled genomes (MAGs) for accurate *N_e_* estimation remains an unresolved issue. To address this, we first focused on the *Thermococcus* genus, a hyperthermophilic archaeon with mutation rate measured recently in our previous analysis (Gu et al., 2021). A large number of public isolates and MAG genomes makes this genus an ideal lineage to assess the performance of MAGs in *N_e_* estimation. Despite the unbiased phylogenetic distribution and comparable internal average nucleotide identity (ANI) between isolate populations (solely consists of isolates) and MAG populations (solely consists of MAGs), the *N_e_* estimates derived from MAGs were significantly smaller than those based on isolates. The underestimation of *N_e_* in MAG populations was also found in three other microbial lineages. These results emphasize the imperative for caution when employing MAGs in evolutionary studies that demand high-quality genomic data.

## 2 Materials and methods

### 2.1 Construction of phylogenomic tree

A comprehensive dataset of 95 genome assemblies affiliated with the *Thermococcus* genus (taxon ID 2263) was downloaded from the NCBI database as of October 2022. This collected dataset included 66 isolates and 29 metagenome-assembled genomes (MAGs). Phylogenomic analysis was conducted based on 53 single-copy genes (AR53), which are universally conserved in the Archaea domain and have undergone minimal recombination events (Parks et al., 2017). The gene sequence extraction, alignment, and trimming were performed using the ‘classify_wf’ function in GTDB-tk v1.7.0 (Chaumeil et al., 2019) with default parameters. Subsequently, the phylogenomic tree was generated using IQ-TREE v2.2.0 (Minh et al., 2020) with the ‘-m MFP’ option, which employs the ModelFinder (Kalyaanamoorthy et al., 2017) to determine the best-fit substitution model (LG + R10 in this study). The phylogeny was rooted using the last common ancestor (LCA) of *T. litoralis* and *T. sibiricus* according to the reference Archaea tree in the GTDB database (release207) and visualized using iTOL (Letunic and Bork, 2021).

### 2.2 Delineation of population boundary

The boundary of panmictic populations was delineated using PopCOGenT (Arevalo et al., 2019) in the ‘single-cell’ mode to account for the incompleteness of MAGs. PopCOGenT functions on the principle that recent homologous recombination events can eliminate single nucleotide polymorphisms (SNPs), thereby generating identical genomic regions between donor and recipient. Consequently, genomes subject to frequent gene transfers are expected to manifest an enrichment of identical genomic regions as opposed to an accumulation of SNPs in genomes devoid of recent gene flow. This gene flow prediction relies on pairwise genome comparisons, making PopCOGenT a suitable tool for analyzing partial genome sequences. As PopCOGenT was designed for closely related genomes at approximately species level (Arevalo et al., 2019), the public genomes were first grouped into preliminary clusters where members share pairwise ANI no less than 90%, a threshold below which genomes were less likely to be assigned to the same species (Tsementzi et al., 2016). We employed dRep v3.4.0 (Olm et al., 2017) with ‘-pa 0.90 -ps 0.90’ settings to predict preliminary clusters, which were then used for population delineation. According the PopCOGenT, genomes exhibiting nucleotide diversity below 0.0355% should be classified into a single clonal complex due to insufficient mutations to identify homologous recombination events (Arevalo et al., 2019). Multiple redundant members within a clonal complex may underestimate the neutral genetic diversity (*π_s_*) and effective population size (*N_e_*) owing to their high genomic identity (Chen et al., 2021; Gu et al., 2021). To avoid underestimation, a single representative genome was retained for each clonal complex. Consequently, only populations comprising at least two non-redundant members were kept for the estimation of π_s_ and *N_e_*.

### 2.3 Estimation of π_s_ and N_e_

The procedures below were conducted for each population individually. To control for potential biases from varying gene prediction algorithms in prior research, protein-coding genes were re-annotated from the collected *Thermococcus* genomes using Prodigal v2.6.3 (Hyatt et al., 2010) with the ‘-p meta’ option. Orthologous gene families were subsequently identified across each population’s genomes using OrthoFinder v2.2.1 (Emms and Kelly, 2019) with default parameters. Single-copy orthologous gene families were aligned at the amino acid level using MAFFT v.7.464 (Katoh and Standley, 2013) and imposed onto the corresponding nucleotide sequences. To minimize the influence of natural selection, π_s_ estimation was performed based on fourfold degenerate sites, which are largely neutral and less affected by natural selection, as identified by the ‘get4foldSites’.^1^ The π_s_ calculation was conducted in accordance with our previous publication (Chen et al., 2021). Finally, the median π_s_ across all single-copy gene families was used to compute *N_e_* based on the equation π_s_ = 2 × *N_e_* × μ, where μ represents the spontaneous mutation rate of type strain *T. eurythermalis* A501 (71.57 × 10^−10^ base substitutions per cell division per nucleotide site) (Gu et al., 2021). The *N_e_* estimation was also carried out based on the mean π_s_ across all single-copy orthologous gene families within each population.

### 2.4 Measurement of genomic and evolutionary features

The sequencing quality and genomic features of the collected *Thermococcus* genomes were assessed using CheckM v1.1.3 (Parks et al., 2015). Taking into account the potential incompleteness and misassembly in MAGs, the estimated genome size was calculated as the assembled genome size divided by the sum of its completeness and contamination (Parks et al., 2017). Within each population, pairwise ANI was calculated using FastANI v1.3 (Jain et al., 2018) with default parameters. To quantify the relative rates and effects of recombination versus mutation, core genome alignments were first executed using Parsnp v1.2 (Treangen et al., 2014) with default parameters for genome assemblies within each *Thermococcus* population. Specifically, MAG populations M-1, M-4, and M-5 were excluded from the Parsnp analysis because the their genomes differ in assembly size over 30% (Treangen et al., 2014). Next, ClonalFrameML v1.1 (Didelot and Wilson, 2015) was implemented to estimate the relative rates and effects of recombination versus mutation for each population.

### 2.5 Comparison of genomic and evolutionary features

To determine the most suitable statistical approach for downstream analyses, either phylogenetically dependent or independent, phylogenetic signals in genomic features and evolutionary attributes were assessed between isolate populations and MAG populations. This was represented using Pagel’s λ calculated using the ‘phylosig’ function in the ‘phytools’ R package (Revell, 2012), with values ranging from 0 to 1 to indicating the absence or presence of a strong phylogenetic signal, respectively. A pronounced phylogenetic signal was observed in GC content, necessitating the use of the phylogenetically dependent ‘phylANOVA’ function in the ‘phytools’ R package (Revell, 2012) for statistical comparison. In contrast, no phylogenetic signal was detected in the population classification or other features. Accordingly, the phylogenetically independent Mann–Whitney U test was employed for comparative analyses for estimated genome size, coding density, internal ANI, *N_e_*, and relative rates and effects of recombination versus mutation.

### 2.6 Updating the analysis with expanded genomic datasets

To determine whether the observed trends in *Thermococcus* are broadly applicable, we expanded our analyses to three bacterial genera: *Flavobacterium*, *Agrobacterium*, and *Lactococcus*. They were selected from the 22 microbial lineages with mutation rate and panmictic populations available (Chen et al., 2021). We excluded other lineages due to either a scarcity of MAG populations or the costly computational demands for analyzing a large number of genomes (over 7,000). The same approaches mentioned above were employed for each lineage to delineate their populations, estimate *N_e_*, and measure and compare genomic and evolutionary features. The only change in this step was the replacement of AR53 with 120 bacterial single-copy genes (BAC120) (Parks et al., 2017) during the construction of phylogenomic trees.

## 3 Results

### 3.1 Genome sampling and population delineation

**Figure 1:**
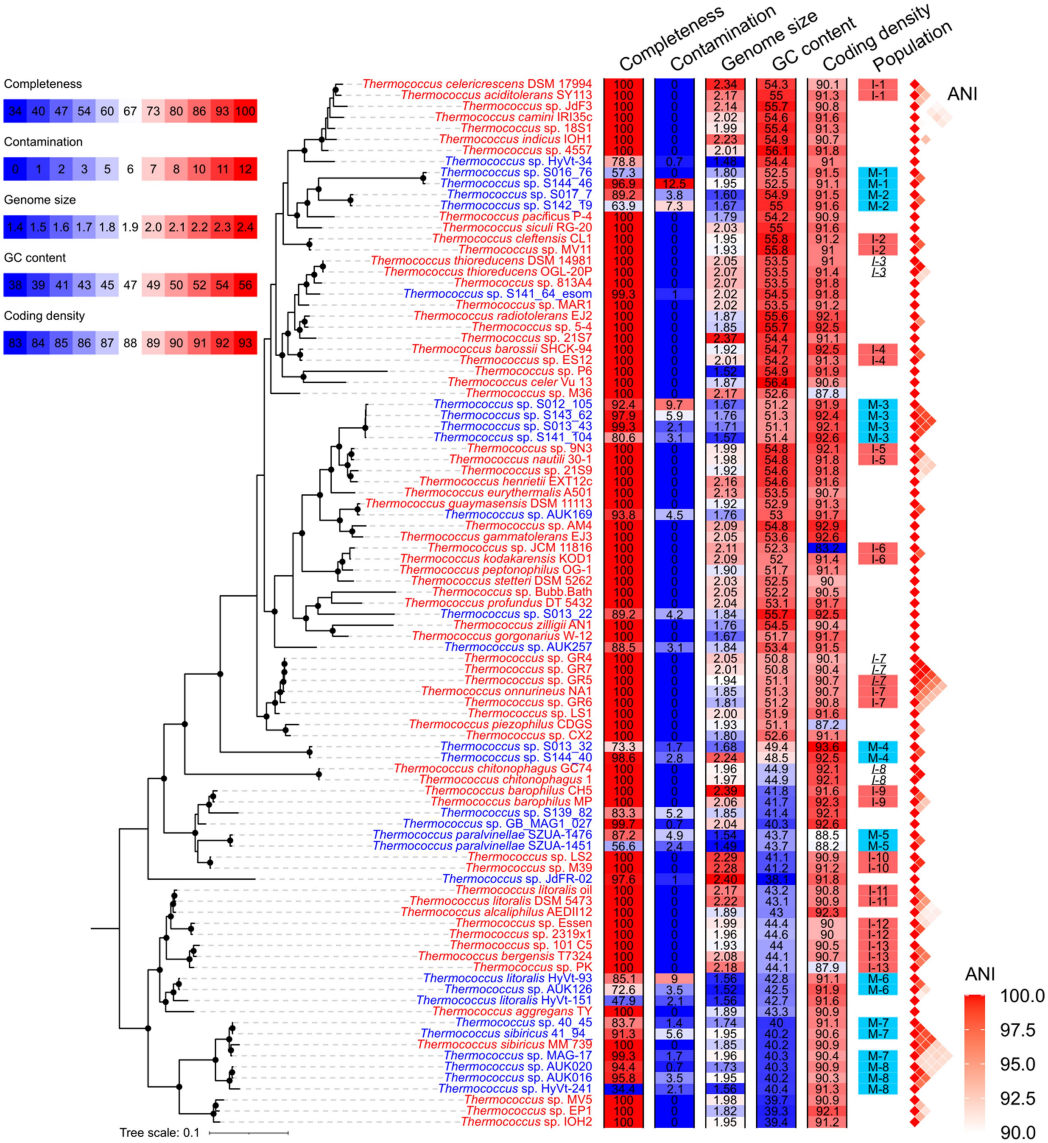
Phylogenetic topology of *Thermococcus* genomes. The phylogenomic tree was constructed based on 53 conserved single-copy genes in Archaea (AR53) using GTDB-tk and IQ-TREE. Nodes supported by bootstra*p* values exceeding 95% are denoted by solid circles in the phylogenomic tree. The genome IDs of isolates and MAGs are colored in red and blue, respectively. Genomic features including completeness, contamination, estimated genome size, GC content, and coding density, are shown using heatmaps after the phylogeny. The legend for these heatmaps is located at the upper-left corner. Populations delineated by PopCOGenT, comprising at least two members, are annotated adjacent to the heatmaps. Isolate populations and MAG populations used for *N_e_* estimation are marked with red and blue shades, respectively. Within each clonal complex, members are indicated by italicized population IDs. Genomes excluded from *N_e_* estimation are not shaded. Pairwise ANI over 90% are shown on the right of populations.

**Figure 2:**
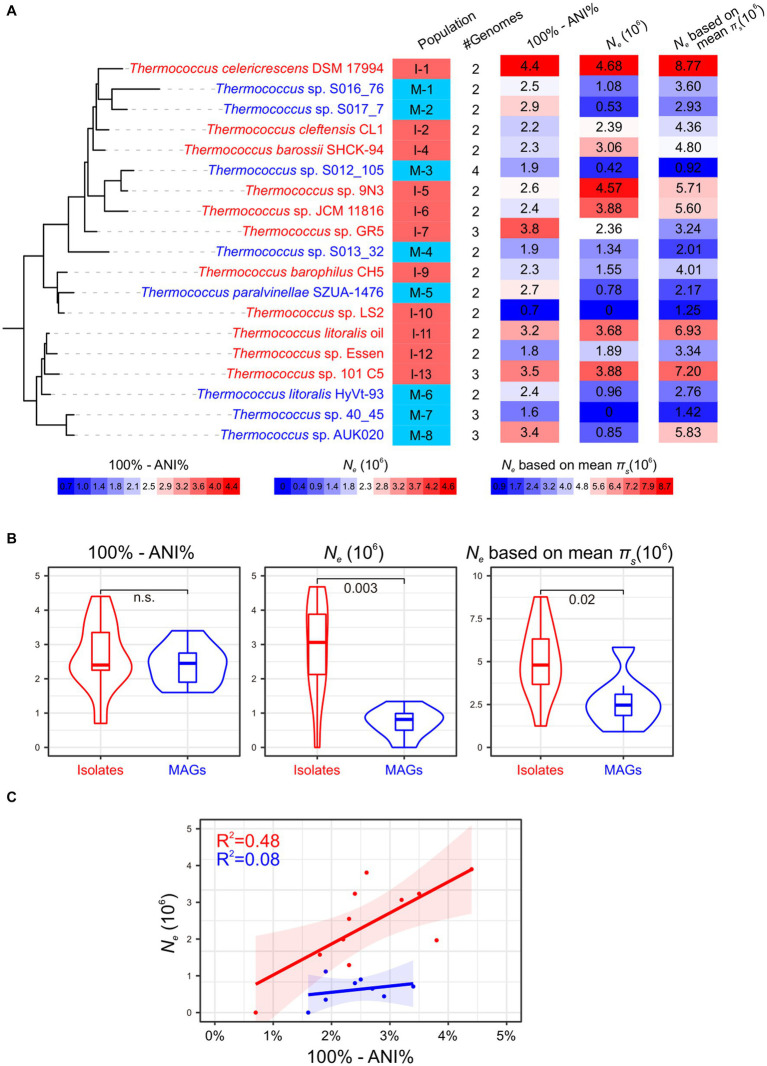
Impact of MAGs on *N_e_* estimation. Isolate populations and MAG populations are colored in red and blue, respectively. **(A)** The *N_e_* estimation for 19 *Thermococcus* populations, excluding populations I-3 and I-8 due to their composition solely of clonal complexes. The phylogenetic tree is pruned from that is shown in Figure 1, with genome IDs from one representative genome annotated. Population IDs and the number of genomes with each population are indicated. The ‘100% - ANI%’ depicting genetic diversity within each population and *N_e_* estimates are shown using heatmaps after the phylogeny. The legend for these heatmaps is located below the phylogeny. **(B)** Comparison of whole genomic divergence (100% - ANI%) and *N_e_* between isolate populations and MAG populations as shown in violin plots. Statistic comparisons are conducted using Mann–Whitney U test due to the lack of phylogenetic signals (Pagel’s λ = 0). Non-significant *p* values (*p* > 0.05) are marked as n.s… *N_e_* estimations based on the median and mean π_s_ across all single-copy orthologous gene families are presented in the middle and right panels, respectively. **(C)** The relationship between *N_e_* and whole genomic divergence (100% - ANI%) in both isolate populations and MAG populations.

A total of 66 isolated strains and 29 metagenome-assembled genomes (MAGs) affiliated with the *Thermococcus* genus were collected from the NCBI database (Supplementary Table S1). Of these MAGs, 24 exhibited genomic completeness exceeding 50% and contamination below 5% as assessed by CheckM, thus meeting the criteria for medium or high sequencing quality (Bowers et al., 2017). This number was refined to 20 when completeness and contamination were evaluated using a customized gene set comprising 289 single-copy orthologous gene families shared across all genomes of isolates (Figure 1 and Supplementary Table S1). The 95 *Thermococcus* genomes were partitioned into 66 distinct populations by PopCOGenT. Among these, 45 populations were constituted of only one genome, while the remaining 21 populations each comprised two to four genomes (Figure 2A). Notably, three populations were identified as harboring clonal complexes (see the Materials and Methods section). To mitigate the potential bias in *N_e_* estimation induced by these clonal complexes, only one representative genome was retained from each complex for subsequent analyses. This led to the exclusion of two entire populations (I-3 and I-8) consisting solely of clonal complexes. Additionally, one genome (*T.* sp. GR5) was retained as a representative in population I-7, while *T.* sp. GR4 and *T.* sp. GR7 were discarded as they form complex together with *T.* sp. GR5. As a result, 19 populations were retained for downstream analyses (Figure 2A and Supplementary Table S2). Among them, 11 are isolate populations and eight are MAG populations.

### 3.2 Impact of MAGs on *N_e_* estimation

The 19 *Thermococcus* populations were uniformly distributed across the phylogenomic tree (Pagel’s λ = 0; Figure 2A), suggesting an anticipated congruence in genomic and evolutionary characteristics between isolate populations and MAG populations. The internal average nucleotide identity (ANI) within each population ranges from 95.6 to 99.3% (Supplementary Tables S2,S3), conforming to the operational criteria for defining a prokaryotic species at a 95% ANI threshold (Jain et al., 2018). Given that ANI serves as an indicator of genomic divergence across both fourfold degenerate sites and other genomic regions, it is posited to exhibit a positive correlation with neutral diversity and *N_e_* under certain conditions (Schloissnig et al., 2013). No significant difference was observed in the whole genomic divergence, which was represented by 100% - ANI%, between isolate populations and MAG populations (Figure 2B), thereby facilitating the comparability of *N_e_* estimation across these population categories.

Variability in microbial mutation rates can occur in different species even within the same genus. For instance, mutation rates in *Vibrio cholerae, V. fischeri, and V. shilonii* have been estimated to range from 1.07 × 10^−10^ to 2.29 × 10^−10^ base substitutions per cell division per site (Dillon et al., 2017; Strauss et al., 2017). However, in the absence of mutation rate data for other *Thermococcus* lineages, we utilized a spontaneous mutation rate of 71.57 × 10^−10^ base substitutions per cell division per site from *T. eurythermalis* A501 as a representative value for all *Thermococcus* populations (Gu et al., 2021). Initial *N_e_* estimations were derived using the median value of neutral genetic diversity (π_s_) across all single-copy orthologous gene families within each population. In this case, two populations, I-10 and M-7, exhibited zero *N_e_* due to the absence of neutral genetic diversity in over half of their gene families. The remaining ten isolate populations displayed estimated *N_e_* values ranging from 1.55 × 10^6^ to 4.68 × 10^6^ (Figure 2), consistent with our prior estimates (4.22 × 10^6^) based on seven *T. eurythermalis* genomes with an internal ANI of 95.4% (Chen et al., 2021; Gu et al., 2021). Surprisingly, the estimated *N_e_* values for the remaining seven MAG populations varied from 0.42 × 10^6^ to 1.34 × 10^6^, significantly lower than those of isolate populations (Mann–Whitney U test, *p* = 0.003). This pattern persisted when *N_e_* was calculated using mean π_s_ values (Figure 2B). Moreover, a positive correlation between *N_e_* and whole genomic divergence (100% - ANI%) was observed in isolate populations (R^2^ = 0.48, *p* = 0.01; Figure 2C) as expected (Schloissnig et al., 2013). However, this correlation was absent in MAG populations (R^2^ = 0.08, *p* = 0.5), raising questions about the reliability of incorporating MAGs in *N_e_* estimations.

To assess the consistency of the aforementioned *Thermococcus* findings across other prokaryotic lineages, the same analyses were conducted for three bacterial genera: *Flavobacterium*, *Agrobacterium*, and *Lactococcus*. A total of 985, 546, and 625 genome assemblies were downloaded from the NCBI GenBank for each lineage (Supplementary Table S4). Applying the same criteria, we categorized *Flavobacterium* genomes into 40 isolate populations and 15 MAG populations, *Agrobacterium* genomes into 23 isolate populations and five MAG populations, and *Lactococcus* genomes into 13 isolate populations and 10 MAG populations (Supplementary Table S5). The number of genomes within these populations ranges from two to 136, with a median of two genomes (Supplementary Table S5).

**Figure 3:**
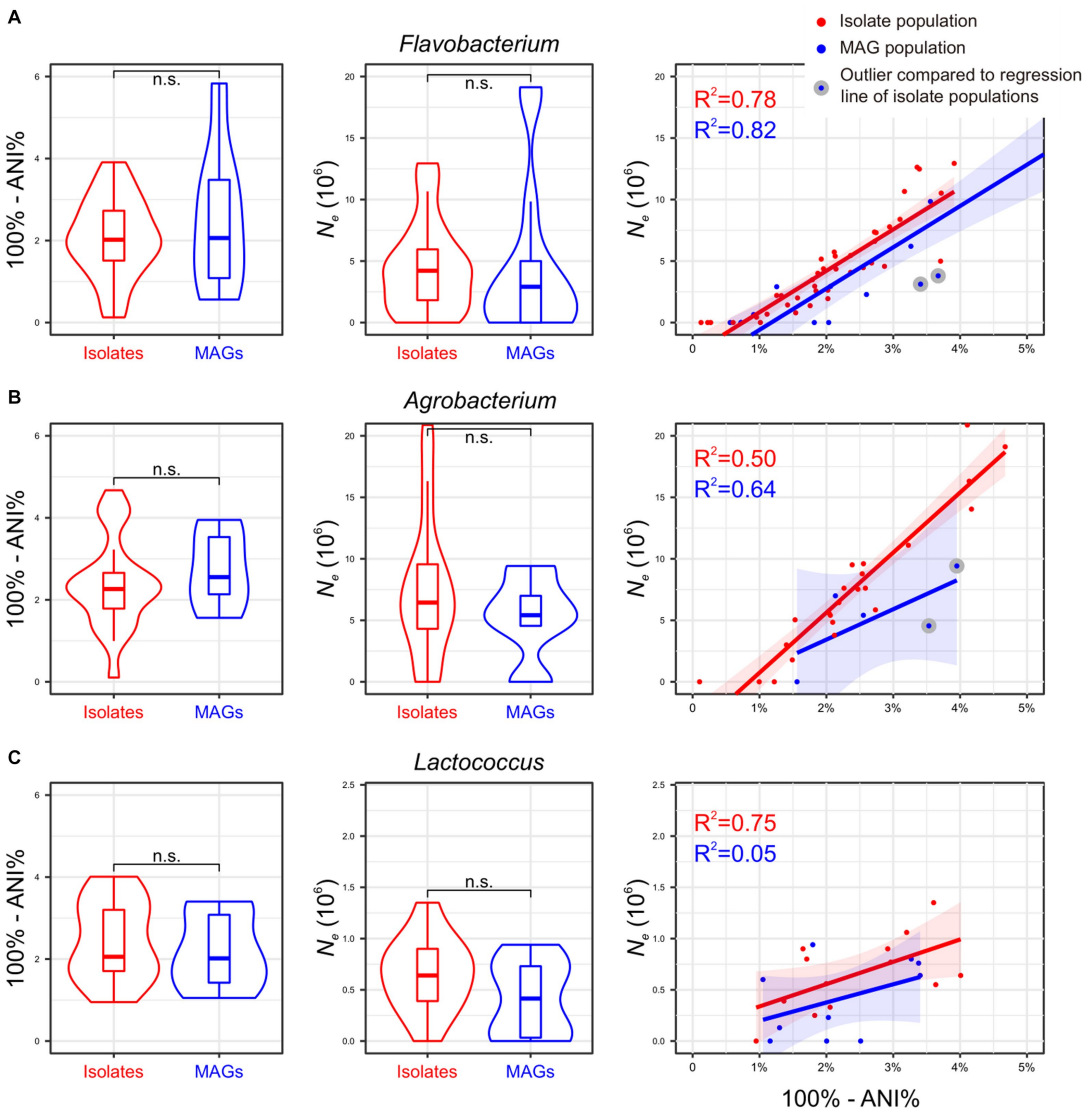
**(A–C)** Impact of MAGs on *N_e_* estimation using three additional bacterial genera. The results mirror those in Figure 2, adapting it to *Flavobacterium*, *Agrobacterium*, and *Lactococcus* genera. The corresponding phylogenetic trees are shown in Supplementary Figure S1.

Consistent with *Thermococcus*, both isolate populations and MAG populations in *Agrobacterium* and *Lactococcus* lineages are evenly distributed across the phylogenomic tree (Pagel’s λ = 0 for *Agrobacterium* and *Lactococcus*; Supplementary Figure S1), while a weak phylogenetic signal in the population distribution was found in *Flavobacterium* lineage (Pagel’s λ = 0.4). Furthermore, the whole genomic divergence (100% - ANI%) did not exhibit significant differences between isolate populations and MAG populations in each lineage. The mean value of *N_e_* estimates derived from MAG populations are slightly lower than those from isolate populations across all three lineages, although this difference does not reach statistical significance (Figure 3). Of particular interest, there is a positive correlation between the whole genomic divergence (100%-ANI%) and *N_e_* estimates in isolate populations in all four investigated genera (Figure 2C, 3), which could be used to determine an expected *N_e_* value given a genomic divergence level. However, both *Flavobacterium* and *Agrobacterium* exhibit two MAG populations each, displaying *N_e_* estimates that significantly deviated from such regression line (Figures 3A,B). This finding suggests that these four MAG populations may tend to underestimate *N_e_* compared to their corresponding isolate populations at similar levels of genetic divergence, underscoring the limitations of MAGs in accurately estimating *N_e_*.

### 3.3 Comparison of other genomic and evolutionary features between isolate and MAG populations

As mentioned earlier, isolate populations and MAG populations were expected to exhibit comparable genomic attributes due to their even distribution across the phylogenomic trees (Figure 2A and Supplementary Figure S1). To further assess the utility of MAGs in ecological studies, we conducted a comparative analysis of estimated genome size, GC content, and coding density between these two population categories. The estimated genome size was significantly smaller in MAG populations affiliated with *Thermococcus* and *Flavobacterium* lineages (Supplementary Figure S2). Additionally, a significantly lower coding density was observed in *Flavobacterium* and *Agrobacterium* lineages (Supplementary Figure S2). These results highlighted a systematic bias in genomic features in MAGs, a concern that that may have broader implications for metagenomic studies (Meziti et al., 2021).

MAGs have been previously demonstrated to provide robust estimates of microbial recombination rates and effects, akin to those derived from isolates in a recent investigation targeting *Prochlorococcus* and freshwater clade LD12 (Ngugi et al., 2023). Our study corroborated this observation, revealing that the relative frequency (*ρ/θ*) and effect (*r/m*) of recombination to mutation in each of the four lineages were largely congruent between MAG populations and isolate populations (Supplementary Figure S2). Nevertheless, it should be noted that the generalizability of this pattern to other microbial lineages remains an open question. Given the inherent risk of chimeric assembly in MAGs (Taş et al., 2021), exercising caution is recommended when employing MAGs for evolutionary estimations.

## 4 Discussion

Metagenomic sequencing provides an important resource in ecological research, shedding light on both microbial diversity and metabolic capabilities (Sunagawa et al., 2015). Despite its utility, the suitability of MAGs for evolutionary analyses, which frequently depend on high-quality genome assemblies and nucleotide-level diversity, remains an open question. In this study, we assessed the performance of MAGs in estimating *N_e_*, which is an important parameter in population genetics determining the relative contribution of natural selection and genetic drift. Our analysis of the *Thermococcus* revealed a significant underestimation of *N_e_* in MAG populations compared to their isolate counterparts. Similar trends were observed in the *Flavobacterium* and *Agrobacterium* lineages. These two genera each possess two MAG populations showing significantly lower *N_e_* values than isolate populations showing comparable genomic divergence, which would normally indicate similar levels of neutral diversity (*N_e_*). These findings indicate that MAGs are not able to ensure an accurate estimation of *N_e_*, underscoring the need for caution in their use for evolutionary research.

The biased *N_e_* estimation may be a result of the inherent limitations of MAGs. These include the loss of allele frequency information during the metagenomic assembly process and the inability to retrieve highly variable genomic fragments during the binning process. On one hand, MAGs typically represent the most abundant allelic variants in the wild population (Crits-Christoph et al., 2020), so they often fail to capture rare variants from the environments. This omission likely results in an insufficient representation of population diversity, contributing to the underestimation of *N_e_*. On the other hand, MAGs are often characterized by the absence of genomic fragments subjected to frequent gene flows, such as prophages, plasmids, and other mobile elements (Meziti et al., 2021). These fragments usually exhibit accelerated evolutionary rate and larger genetic variation (Rodríguez-Beltrán et al., 2021), and their absence in MAGs may further skew *N_e_* estimates downward. This is also evidenced by the smaller genome sizes estimated in MAGs, based on a set of conserved core genes (Parks et al., 2015).

The challenges highlighted in above may be partially overcome by the advancements in metagenomic methodologies. First, metagenomic read recruitment is a promising approach to capture more genetic diversity in wild populations. Indeed, the existing computational tools based on metagenomic recruitment have been successfully applied to investigate microbial recombination (Lin and Kussell, 2019) and nucleotide diversity (Olm et al., 2021) from environmental samples, thereby providing valuable insights especially in extreme habitats where microbial isolates are challenging to obtain (Peng et al., 2023). Nevertheless, the related tools for *N_e_* estimation remain to be further developed, with several challenges remains. One major issue is accurately defining population boundaries and determining appropriate read mapping similarity (Nowinski et al., 2023), which can vary across genes within the same population, especially in complex environmental communities (Bahram et al., 2018). Another limitation is the dependency on high-quality reference genomes, necessitating improved culturing techniques and a broader collection of isolate genomes (Xian et al., 2020). Second, the completeness and quality of MAGs could be improved by hybrid metagenomic assembly that incorporating long-read and short-read data (Bertrand et al., 2019). Such high-quality MAGs could also serve as reference genomes for recruitment-based methods. In addition to isolates and MAGs, single amplified genomes (SAGs) offer an alternative, though more costly, genomic resource for *N_e_* estimation, generally yielding reliable results akin to isolated genomes (Chen et al., 2021). In summary, MAGs are not able to estimate *N_e_* accurately based on the existing tools, while isolates and SAGs are more reliable for this purpose. Nevertheless, the potential for refining methodologies to improve *N_e_* estimation using metagenomic data warrants further exploration.

## Data availability statement

The original contributions presented in the study are included in the article/Supplementary material, further inquiries can be directed to the corresponding author.

## Author contributions

XW: Formal analysis, Funding acquisition, Investigation, Writing – review & editing. XF: conceptualization, funding acquisition, writing original draft, review and editing.

## Footnotes

^1^ https://github.com/brunonevado/get4foldSites
