# Supplementary material for: Challenges in estimating effective population sizes from metagenome-assembled genomes
Source: Front Microbiol. 2024 Jan 5;14:1331583. doi: 10.3389/fmicb.2023.1331583 (PMC10797056; doi:10.3389/fmicb.2023.1331583)

Fig. S1. Phylogenomic trees for populations used for ne estimation across three bacterial genera: *Flavobacterium* (A), *Agrobacterium* (B), and *Lactococcus* (C). The genome IDs from isolate and MAG populations are colored in red and blue, respectively.

A

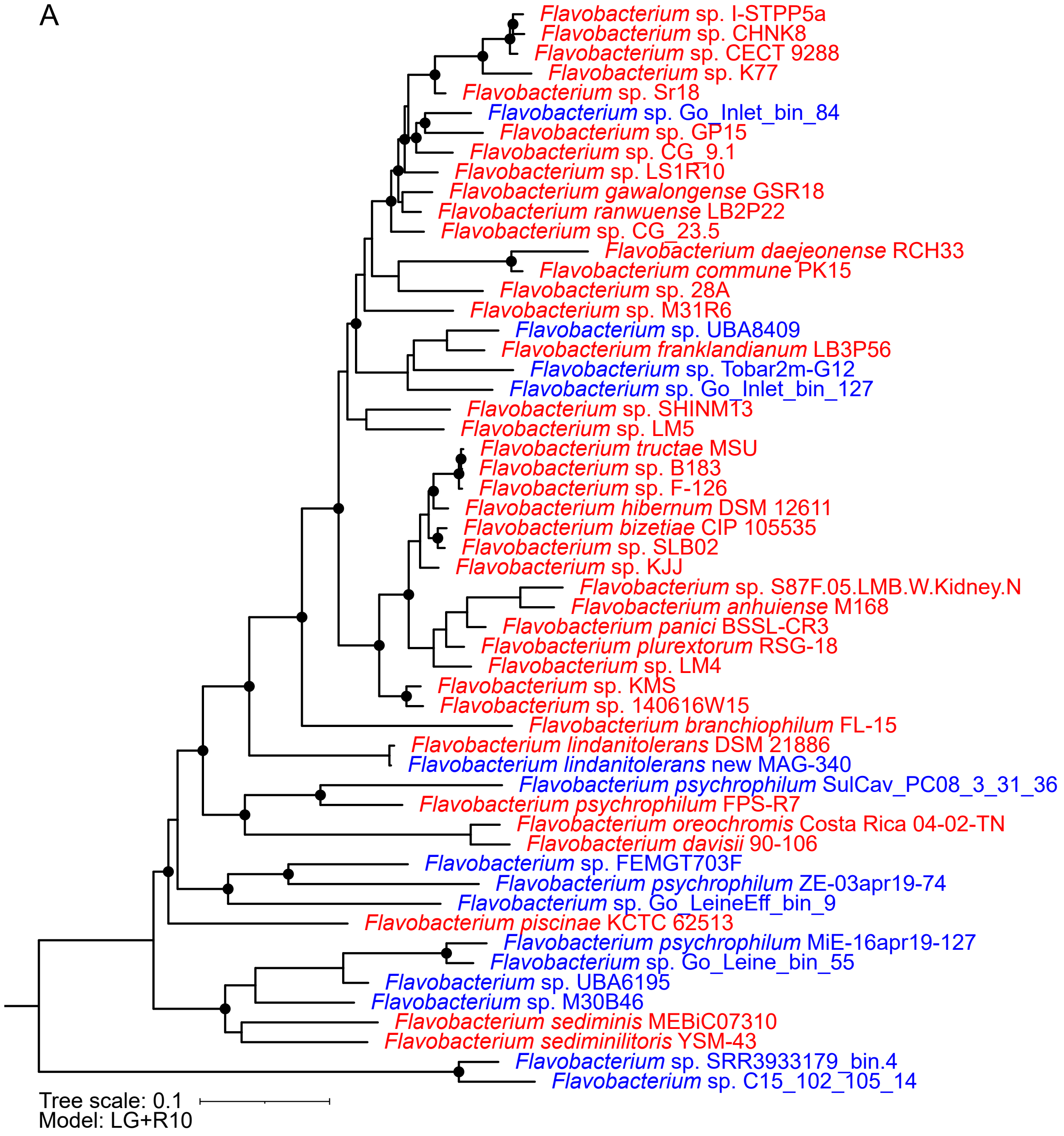

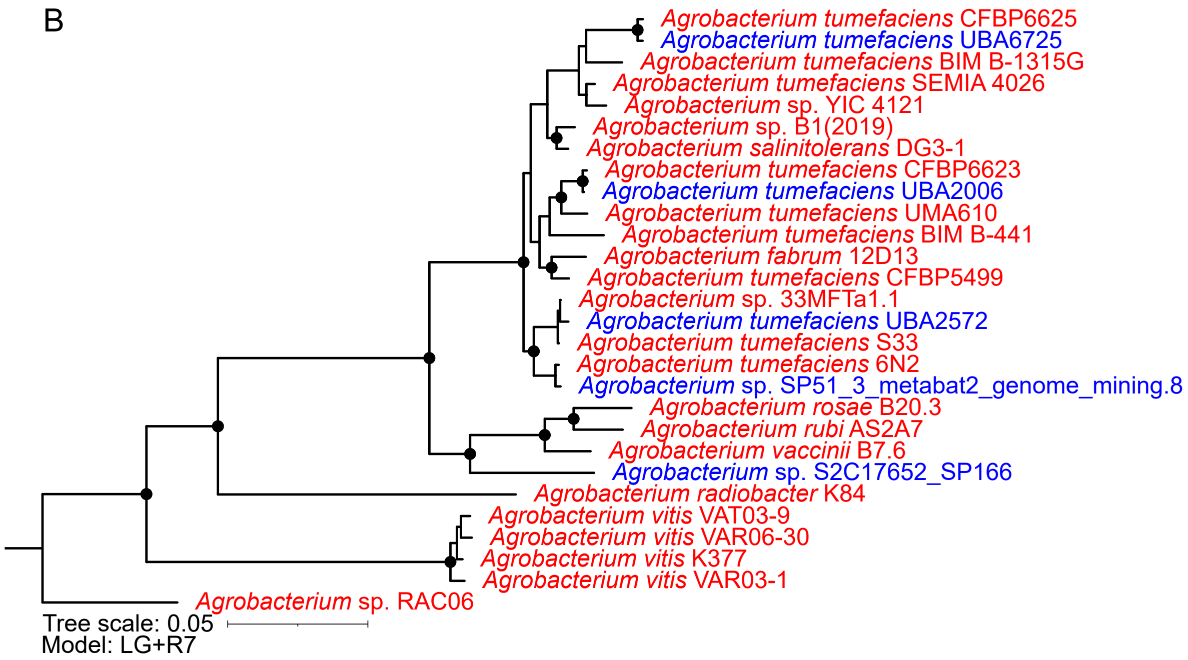
B
Agrobacterium tumefaciens CFBP6625
Agrobacterium tumefaciens UBA6725
Agrobacterium tumefaciens BIM B-1315G
Agrobacterium tumefaciens SEMIA 4026
Agrobacterium sp. YIC 4121
Agrobacterium sp. B1(2019)
Agrobacterium salinitolerans DG3-1
Agrobacterium tumefaciens CFBP6623
Agrobacterium tumefaciens UBA2006
Agrobacterium tumefaciens UMA610
Agrobacterium tumefaciens BIM B-441
Agrobacterium fabrum 12D13
Agrobacterium tumefaciens CFBP5499
Agrobacterium sp. 33MFTa1.1
Agrobacterium tumefaciens UBA2572
Agrobacterium tumefaciens S33
Agrobacterium tumefaciens 6N2
Agrobacterium sp. SP51_3_metabat2_genome_mining.8
Agrobacterium rosae B20.3
Agrobacterium rubi AS2A7
Agrobacterium vaccinii B7.6
Agrobacterium sp. S2C17652_SP166
Agrobacterium radiobacter K84
Agrobacterium vitis VAT03-9
Agrobacterium vitis VAR06-30
Agrobacterium vitis K377
Agrobacterium vitis VAR03-1
Agrobacterium sp. RAC06
Tree scale: 0.05
Model: LG+R7

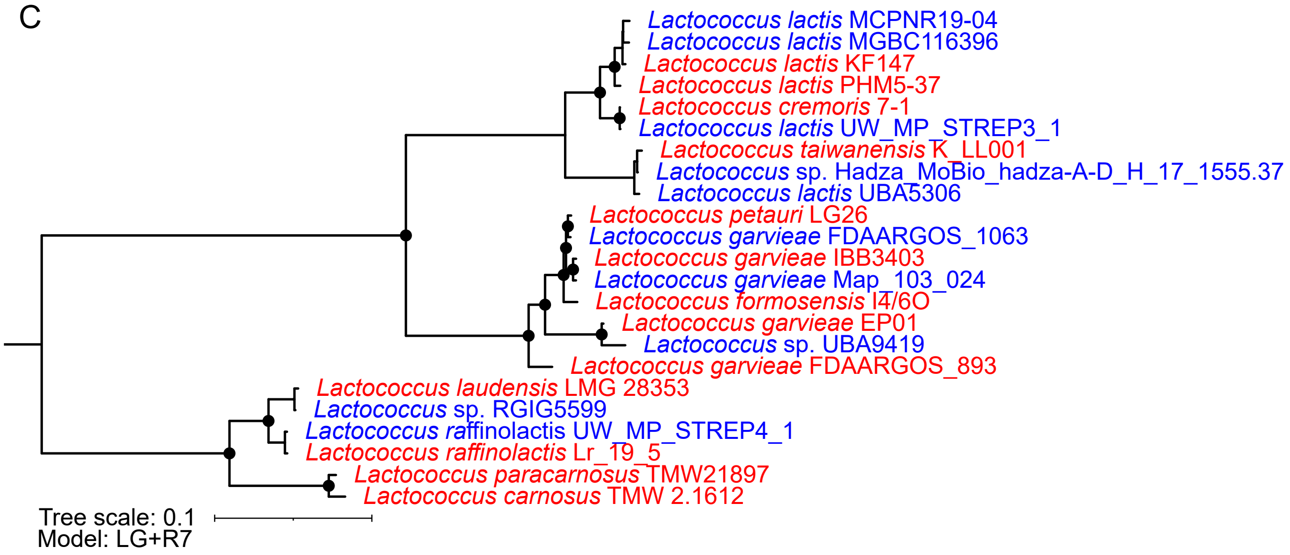
C
Lactococcus lactis MCPNR19-04
Lactococcus lactis MGBC116396
Lactococcus lactis KF147
Lactococcus lactis PHM5-37
Lactococcus cremoris 7-1
Lactococcus lactis UW_MP_STREP3_1
Lactococcus taiwanensis K_LL001
Lactococcus sp. Hadza_MoBio_hadza-A-D_H_17_1555.37
Lactococcus lactis UBA5306
Lactococcus petauri LG26
Lactococcus garvieae FDAARGOS_1063
Lactococcus garvieae IBB3403
Lactococcus garvieae Map_103_024
Lactococcus formosensis I4/6O
Lactococcus garvieae EP01
Lactococcus sp. UBA9419
Lactococcus garvieae FDAARGOS_893
Lactococcus laudensis LMG 28353
Lactococcus sp. RGIG5599
Lactococcus raffinolactis UW_MP_STREP4_1
Lactococcus raffinolactis Lr_19_5
Lactococcus paracarnosus TMW21897
Lactococcus carnosus TMW 2.1612
Tree scale: 0.1
Model: LG+R7

Fig. S2. Comparison analysis of genomic and evolutionary features between isolate populations and MAG populations.

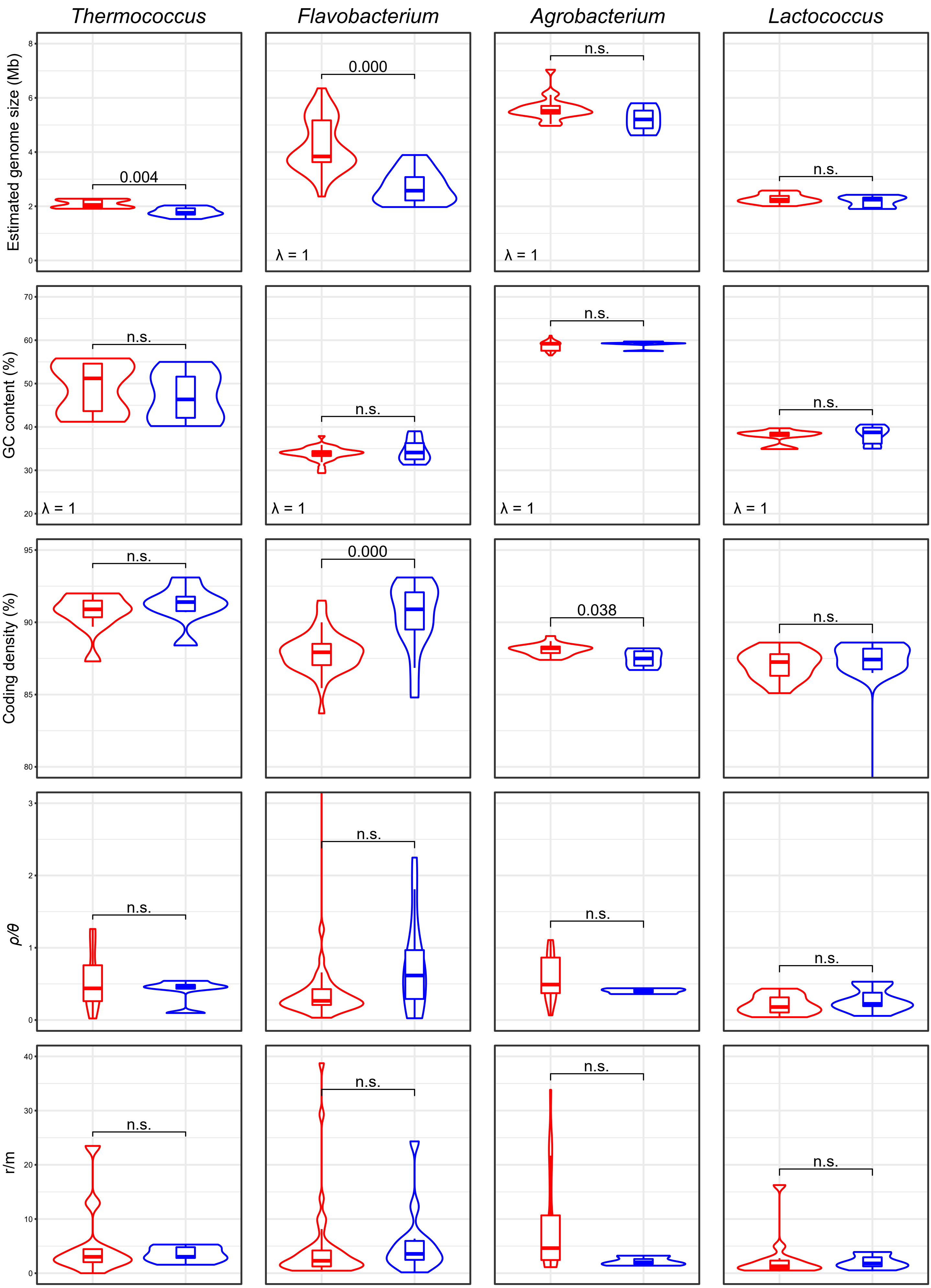

Supplement: Supplementary file 1 [file Data_Sheet_1.pdf]
